# Urticaria After Lidocaine Use for Pecto-Intercostal Nerve Block

**DOI:** 10.7759/cureus.34834

**Published:** 2023-02-10

**Authors:** Vats T Ambai, Neel Atawala, Arun Kalava

**Affiliations:** 1 Department of Anesthesiology, Emory University School of Medicine, Atlanta, USA; 2 Graduate Medical Education, Northside Hospital Gwinnett, Lawrenceville, USA; 3 Medical Education, Mercer University School of Medicine, Savannah, USA; 4 Department of Anesthesiology/Pain Medicine, University of Central Florida College of Medicine, Orlando, USA

**Keywords:** urticaria, case report, allergy, regional anesthesia, lidocaine

## Abstract

Urticaria within one hour of lidocaine injection is a sign of type I (immediate) hypersensitivity to lidocaine, yet most patients suspected of having a lidocaine allergy do not exhibit urticaria. Aside from being a sign of a rare lidocaine allergy, urticaria can also be a symptom of COVID-19. COVID-19 patients who experience urticaria after receiving lidocaine require careful evaluation to determine the cause. Here, we present a case of a patient exhibiting urticaria one hour after a lidocaine injection for the Pecto-intercostal nerve block to treat COVID-19-induced costochondritis.

## Introduction

True immunologically mediated allergic reactions to lidocaine are rare and represent only 1% of all adverse reactions [1]. A commonly seen symptom of true allergic hypersensitivity to lidocaine is an urticarial rash [1-4]. Rashes such as these are not specific to lidocaine allergies. They have been documented in various other disease processes, including cases of COVID-19, obscuring the determination of whether the patient is experiencing a reaction to lidocaine or suffering from the residual effects of a nonallergic process [5,6]. This is a case report of a patient experiencing urticaria one hour after an injection of lidocaine for a Pecto-intercostal nerve block to treat COVID-19-induced costochondritis.

## Case presentation

**Figure 1:**
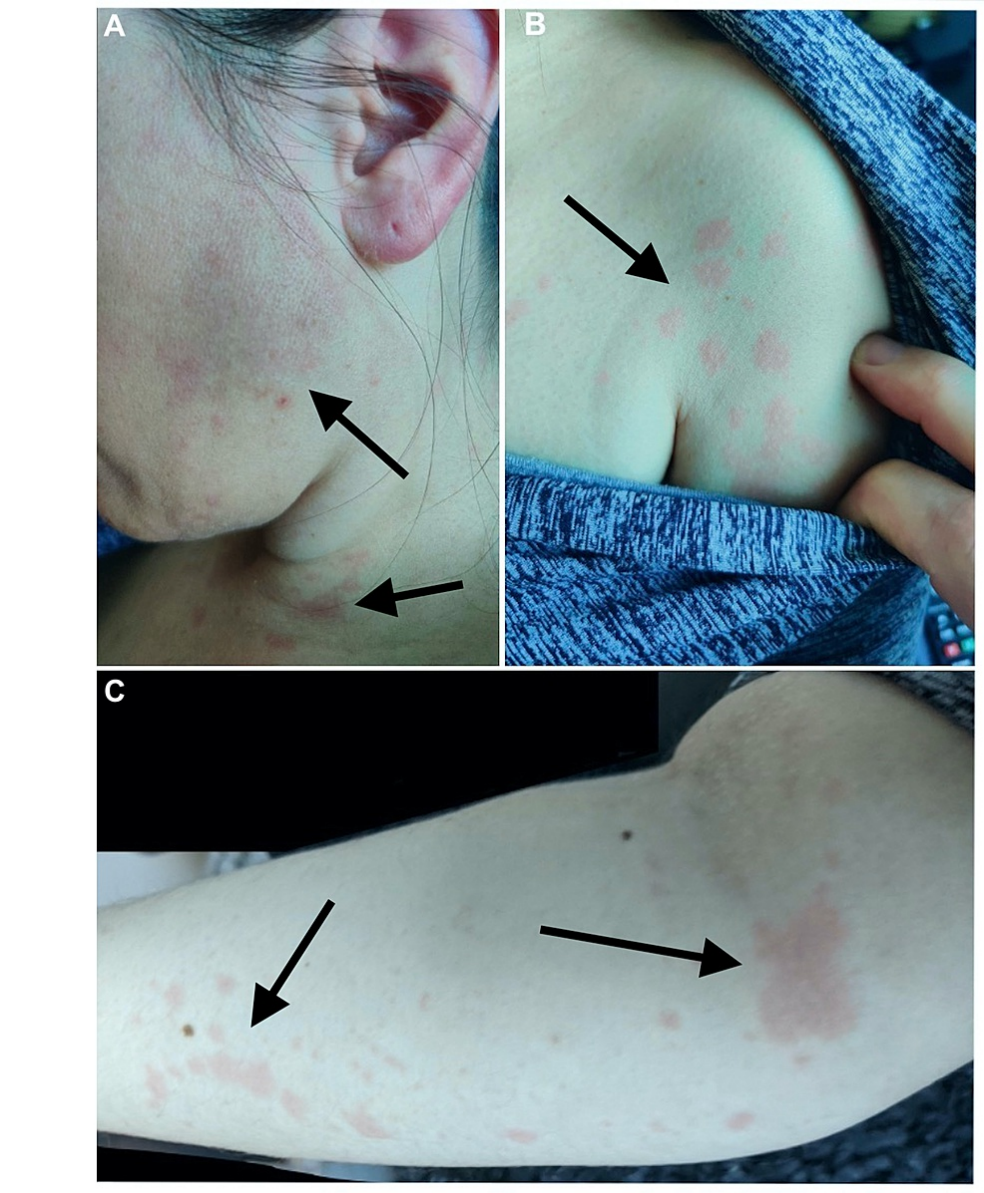
Skin findings one hour after left Pecto-intercostal lidocaine injection. (A) left face, (B) left shoulder, (C) left forearm.

Consent to share information regarding this case was obtained directly from the patient. A 38-year-old female with no significant past medical history was diagnosed with COVID-19 in March 2020 and developed left-sided pleurisy and costochondritis five weeks later. This resulted in shortness of breath with exertion, prompting clinical evaluation. Physical exams, multiple laboratory tests, and diagnostic investigations, including pulmonary function tests, chest X-ray, CT angiogram, and xenon gas MRI revealed no findings. The patient continued to experience significant discomfort and presented for evaluation at a transitional pain clinic. Her allergy history was significant only for cat dander, which caused conjunctival injection and wheezing. She had no history of acute or chronic urticaria. She had received lidocaine twice in the past, once for a dental procedure and another time for the removal of plantar warts, both of which were uneventful. Family history of allergic reactions included a father with an allergy to sulfa-containing drugs and a mother with an allergy to cat dander. She had no relevant psychosocial history. During her treatment at the transitional pain clinic, she consented to a left Pecto-intercostal nerve block. This procedure occurred one year after her COVID-19 diagnosis and was performed after cleaning the injection sites with alcohol and using 15 mL of preservative-free 1% lidocaine without epinephrine as the injectate. At no point during the procedure was the patient exposed to latex, chlorhexidine, antibiotics, non-steroidal anti-inflammatory drugs, steroids, opiates, or additives in the lidocaine solution. The patient tolerated the procedure well and experienced no adverse events in the immediate period following the injection. She was subsequently discharged home and developed a skin rash one hour after injection. The rash appeared as numerous, slightly elevated, non-pruritic, erythematous wheals with irregular borders spanning 1-3 cm in length. Her skin findings spanned the left side of her body, extending across her arm, shoulder, chest, neck, jaw, and cheek (Figures 1A-1C). The rash lasted one to three hours after the initial lidocaine injection. It did not have associated pruritus, edema, changes in vital signs, anxiety, perioral numbness or tingling, dizziness, sympathetic stimulation signs, psychomotor reactions, vasovagal symptoms, or other signs of systemic toxicity. Because this reaction occurred at home, the patient could not be monitored for changes in vital signs. She reported that the rash resolved on its own after three hours without using any medications. 

An allergen-skin test to guide future local anesthetic administration was recommended. Upon follow-up several months later, no allergen testing was completed. However, the patient had received lidocaine in a different medical setting without experiencing any type of urticarial rash, anaphylactic symptoms, or vasovagal symptoms.

## Discussion

In this case, a patient who was treated with a left Pecto-intercostal nerve block using lidocaine developed an urticarial rash one hour after injection. In evaluating this patient’s findings, immediate hypersensitivity (type I), delayed hypersensitivity (type IV), nonallergic hypersensitivity, and IgE-mediated response to COVID-19 infection were all considered. Immediate hypersensitivity reactions occur within one hour and are associated with urticaria in locations away from the lidocaine injection site [1]. They typically occur upon subsequent exposure to an allergen and are dose-independent [2]. Delayed hypersensitivity reactions are experienced between two and three days after exposure and have been shown to exhibit skin findings localized to the site of lidocaine injection [1,3]. Nonallergic hypersensitivity reactions often occur on first exposure and can be dose-dependent [2]. Characteristics of nonallergic hypersensitivity reactions include widespread exanthematous rashes, psychomotor reactions, and vasovagal symptoms [1, 3-5]. Urticarial rashes related to COVID-19 typically appear in the prodromal and acute periods of COVID-19 infection and are not currently known to appear one year after infection [6,7].

A previously published case of a 16-year-old female who received a spinal lidocaine injection demonstrated the manner in which lidocaine hypersensitivity may present. The patient developed erythematous plaques away from the injection site on the neck and trunk, as well as mild edema of the eyelids and lips [8]. These symptoms of a hypersensitivity reaction to lidocaine injection appeared within minutes and were later confirmed by positive intradermal skin testing [8]. In another case, a 26-month-old boy underwent a dental procedure involving lidocaine injections into the buccal mucosa for local anesthesia [9]. That patient returned home without complications but later presented to the ED with angioedema at the injection site. Skin prick test with lidocaine was positive after 15 minutes and confirmed a hypersensitivity response to lidocaine. Based on the timing of the case, the patient’s presentation after lidocaine injection, distribution of skin findings, and lack of other systemic symptoms, type I hypersensitivity to lidocaine is a rare consideration. It is more likely that the patient experienced an anaphylactoid urticarial reaction to the lidocaine, as this is known to primarily be a dose-dependent reaction, with cutaneous symptoms being the most common presenting feature [2]. Anaphylaxis and anaphylactoid reactions may both present with elevated histamine and tryptase levels. However, histamine has been shown to decline to baseline within 60 minutes, whereas tryptase may stay elevated between 15 minutes to 3 hours [10]. Thus, it may be more clinically useful to check serum tryptase in the acute setting if a true allergy is suspected [10]. Patients suspected of having adverse reactions to lidocaine should be counseled to avoid the class of amino-amide local anesthetics overall. They may benefit from the use of the amino-ester group of local anesthetics instead [11]. If not possible to avoid using lidocaine and other amino-amides, pre-treatment with a local injection of 1% diphenhydramine has been shown to provide an adequate amount of anesthesia for up to 30 minutes, comparable to 1% lidocaine [11]. Additionally, diphenhydramine does not exhibit cross-reactivity with the “caine” anesthetics, eliminating the risk of allergic reactions [11].

## Conclusions

Symptoms of a true type I hypersensitivity to lidocaine may include urticaria, but the presence of urticaria alone is not diagnostic of a true allergic reaction. When suspected, positive results on skin testing with an allergist can provide a definitive diagnosis. The patient in this case report experienced a rare urticarial reaction after a lidocaine injection that was not challenged with allergen testing. Subsequent administration of lidocaine without the appearance of any allergic symptoms makes it extremely unlikely that she has a true allergy to lidocaine. This case displays the rarity and complexity of attempting to diagnose lidocaine hypersensitivity. To avoid misdiagnosing a patient’s lidocaine-related urticaria, it is essential to complete a thorough history and physical exam and understand the characteristics of a true hypersensitivity reaction.
